# Secreted Phospholipases A_2_ of Snake Venoms: Effects on the Peripheral Neuromuscular System with Comments on the Role of Phospholipases A_2_ in Disorders of the CNS and Their Uses in Industry 

**DOI:** 10.3390/toxins5122533

**Published:** 2013-12-17

**Authors:** John B. Harris, Tracey Scott-Davey

**Affiliations:** 1 Medical Toxicology Centre and Institute of Neurosciences, Faculty of Medical Sciences, Newcastle University, Newcastle upon Tyne NE2 4HH, UK; 2 Experimental Scientific Officer, Electron Microscopy Unit, Faculty of Medical Sciences, Newcastle University, Newcastle upon Tyne NE2 4HH, UK; E-Mail: tracey.davey@ncl.ac.uk

**Keywords:** snakebite, envenoming, venoms, toxins, phospholipases A_2_, neurotoxicity, myotoxicity

## Abstract

Neuro- and myotoxicological signs and symptoms are significant clinical features of envenoming snakebites in many parts of the world. The toxins primarily responsible for the neuro and myotoxicity fall into one of two categories—those that bind to and block the post-synaptic acetylcholine receptors (AChR) at the neuromuscular junction and neurotoxic phospholipases A_2_ (PLAs) that bind to and hydrolyse membrane phospholipids of the motor nerve terminal (and, in most cases, the plasma membrane of skeletal muscle) to cause degeneration of the nerve terminal and skeletal muscle. This review provides an introduction to the biochemical properties of secreted sPLA_2_s in the venoms of many dangerous snakes and a detailed discussion of their role in the initiation of the neurologically important consequences of snakebite. The rationale behind the experimental studies on the pharmacology and toxicology of the venoms and isolated PLAs in the venoms is discussed, with particular reference to the way these studies allow one to understand the biological basis of the clinical syndrome. The review also introduces the involvement of PLAs in inflammatory and degenerative disorders of the central nervous system (CNS) and their commercial use in the food industry. It concludes with an introduction to the problems associated with the use of antivenoms in the treatment of neuro-myotoxic snakebite and the search for alternative treatments.

## 1. Introduction

**Figure 1:**
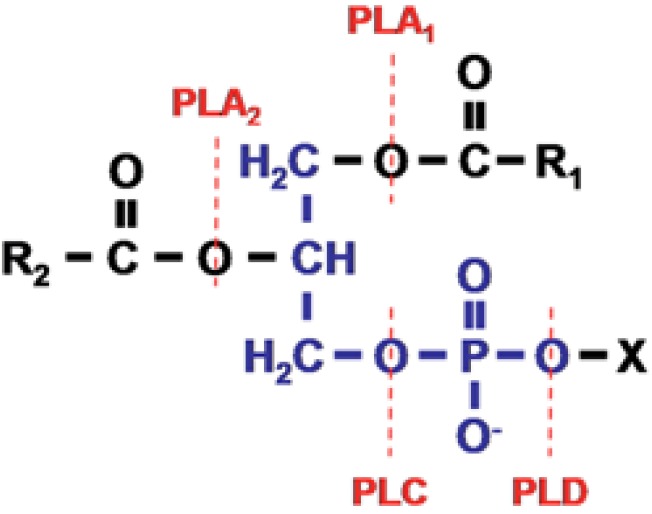
Glycerophospholipid structure and the site of action of phospholipases. The phospholipid molecule consists of a glycerol-3-phosphate (**blue**) esterified at positions sn-1 and sn-2 to non-polar fatty acids. Its phosphoryl group is esterified to a polar head group (x). Phospholipases A_1_ cleave the ester bond at the sn-1 position. Phospholipases A_2_ cleave the ester bond at the sn-2 position. Phospholipases B cleave the ester bonds at both sn-1 and sn-2 positions. Phospholipases C cleave the glycerophosphate bond and phospholipases D remove the polar head group. From The AOCS Lipid Library (http://lipidlibrary.aocs.org/animbio/phospholipases/index.htm).

Phospholipases are enzymes that hydrolyse glycerophospholipids. They fall into one of five principal groups depending on the site at which the glycerophospholipids are cleaved. Phospholipases A_1_ act on the sn-1site, phospholipases A_2_ act at the sn-2 site, phospholipases B act at both sn-1 and sn-2 sites, phospholipases C act on the glycerophosphate bond and phospholipases D remove the polar head group (Figure 1). 

Phospholipases A_2_ (PLA_2_) of snake venoms are the primary concern of this review. PLA_2_s comprise a very large superfamily of enzymes composed of 16 recognised groups within six major types [1,2]. These major types include the secreted PLA_2_s (sPLA_2_), the cytosolic PLA_2_s (cPLA_2_), the calcium independent PLA_2_s (iPLA_2_) the platelet activating factor (PAF) acetyl hydrolase/oxidised lipid lipoprotein associated PLA_2_ (LpPLA_2_s), the adipose PLA_2_s (AdPLA_2_s) and the lysosomal PLA_2_s (LPLA_2_s). The hydrolysis of glycerophospholipids by PLA_2_s results in the release of fatty acid and the production of the relevant lysophospholipid. The enzymes are found in virtually all forms of life from bacteria to invertebrates, vertebrates and plants. They play a major role in the regulation of phospholipid turnover, membrane fluidity and trafficking, cell maturation and maintenance, apoptosis, and the production of the eicosanoids, leukotrienes and prostaglandins. They are of particular interest to the neurologist and neurotoxicologist because sPLA_2_s are intimately involved in the peripheral neuro-myotoxicity caused by envenoming bites by many dangerous snakes and because both s- and cPLA_2_s are implicated in inflammatory and degenerative disease of the CNS [3,4,5,6] PLA_2_s are also widely used in the food processing industry for the refinement of oils and the processing of eggs, cereals and dairy produce (in which role they are not considered to pose any form of health risk) and there is great interest in their possible use for the remediation of oil-contaminated land [7,8,9].

## 2. The Venom-Derived Secreted PLA_2_s

The venom-derived sPLA_2_s fall into four major sub-Types conventionally referred to as Types 1, 2, 3 and 4. Types 1 and 2 sPLA_2_s are found in the venoms of snakes. Type 3 sPLA_2_ enzymes are structurally unique and are found only in the venoms of the Gila Monster (*Heloderma suspectum)* and the Mexican Beaded Lizard (*Heloderma horridum horridum)*, and venom of the bee *Apis mellifera*. Type 4 sPLA_2_ are very small polypeptides of between 40 and 80 residues that are secreted in the venom of some marine cone snails of the genus *Conus*. Type 3 and 4 sPLA_2_s are of considerable biological interest but are not further considered here. 

**Figure 2:**
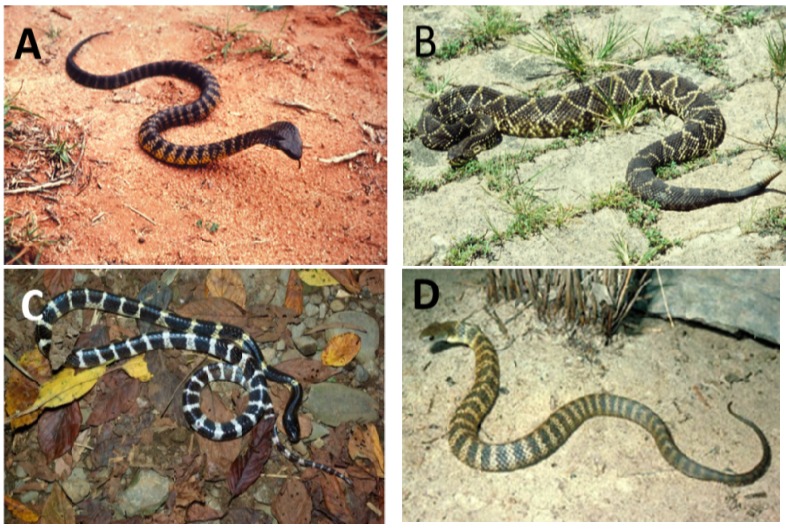
Snakes frequently involved in major neuro-myotoxic envenoming in humans. (**A**) the Australian Tiger snake, *Notechis scutatus* and (**B**) the South American rattlesnake, *Crotalus durissus terrificus*, both cause neurotoxicity and rhabdomyolysis; (**C**) the Taiwanese Multi-banded krait, *Bungarus multicinctus* causes severe neurotoxicity but no myotoxicity; (**D**) the Beaked sea snake, *Enhydrina schistosa*, causes severe myotoxicity but rarely neurotoxicity in human subjects.

The sPLA_2_s of snake venoms were the first phospholipases to be formally characterised. They are found in two major groups of snake—the elapids and sea snakes (New World snakes of principally of SE Asia, Australasia and parts of the Americas) and the vipers and crotalids (principally of the Americas, and Eurasia). The elapids and sea snakes inoculate venom via short, fixed fangs of between 1 and 5 mm in length and include major species of clinical interest such as tiger snakes (genus *Notechis*) and taipans (genus *Oxyuranus*) of Australia and Papua New Guinea, the kraits (genus *Bungarus*) of SE Asia and sea snakes (Figure 2).

The Old World snakes possess a hinged maxillary bone and carry much larger fangs. The hinged maxillary bone enables the fangs to be folded back towards the throat thus enabling the fangs to be accommodated. The difference is sometimes significant clinically because the short fangs characteristic of elapids and sea snakes means that venom is not inoculated into the deep tissues. The much longer fangs of the viperids and crotalids can result in the inoculation of venom into rather deeper tissues. 

The primary structures of numerous venom-derived, pancreatic and other sPLA_2_s have been determined [10]. In terms of primary structure they are typically single chain polypeptides of 115–125 amino acid residues, and a molecular mass of 13–15 kDa. The amino acid sequences of the individual sPLA_2_s can be aligned according to the invariant residues and show high degrees of homology among species but they can be subdivided into distinct groups to take into account differences in the number of disulphide bonds, the presence/absence of an N-terminal heptapeptide, similar to that seen in pancreatic pro-enzymes, and a solitary half cysteine that is involved with the formation of covalently linked hetero-dimers that characterise some of the venom-derived sPLA_2_s such as the β-bungarotoxins. The single chain polypeptides are typically cross-linked by 7 (occasionally 6 or 8) disulphide bonds linking Cys residues between homology positions 11 and 77, 27 and 124, 29 and 45, 44 and 105, 51 and 96, 61 and 91 and 84 and 98. The enzyme is more than 50% α-helix and 10% β-sheet. The active site centres on six residues: His-48, Asp-49, Tyr-28, Gly-30, Gly-32 and Asp-99. This centre is also the binding site for Ca^2+^ which is an obligatory co-factor for the activation of catalytic activity. This basic structure is highly conserved between species. They exhibit little selectivity in terms of the preference for the type of fatty acid at the sn-2 position of the phospholipids. 

Group 1a sPLA_2_s are homologous with the mammalian pancreatic PLA_2_ (Group 1b sPLA_2_) but they differ in the sense that the pancreatic enzyme is secreted as an enzymatically inactive zymogen with an additional 7-residue extension on the N-terminus. On secretion the enzyme is activated by the proteolytic removal of the heptapeptide. 

The sPLA_2_s of the venoms of Old World snakes are structurally similar to the Group 1a sPLA_2_ enzymes but they possess a small, additional, C-terminal tail and a slightly different organisation of disulphide bonds. They are identified as Group 2 sPLA_2_. A sub-group of Group 2 sPLA_2_, identified as Group 2b sPLA_2_, possesses a Lys at homology position 49 rather than Asp. As a result of that substitution they are unable to bind Ca^2+^. These compounds have negligible catalytic activity but retain very high levels of more general cytotoxicity as well as myotoxicity. They are found exclusively in the venoms of viperid snakes. 

A specific, individual, sample of venom from a venomous snake may contain several sPLA_2_ isoforms with differing levels of PLA_2_ activity and differing degrees of toxicity. For example the venom of the elapid, *Notechis scutatus*, contains a major Type 1a toxic sPLA_2_, notexin Np, and a very closely related isoform, notexin Ns, as well as the catalytically active, toxic sPLA_2_, notechis 11-5 and an inactive sPLA_2_ isoform, notechis 11-1. There is no evidence of any form of co-operativity between the isoforms and they can be considered as multiple monomeric isoforms of the enzyme. Other sPLA_2_s are multimeric and between two and five individual monomers associate to form the intact toxin [11]. The nature of the association is variable but in every case there is at least one component that has all the structural and functional features of a monomeric sPLA_2_. For example, the principal toxin of the venom of the Australian taipan, *Oxyuranus scutellatus*, is taipoxin. This is a 1:1:1 complex of three sPLA_2_ isoforms, the basic α-taipoxin, the neutral β-taipoxin (which may comprise two isoforms β1 and β2) and the acidic γ-taipoxin. The individual β- and γ- isoforms are non-toxic and the α-isoform has about 10% of the activity of the full taipoxin complex. The combination of α- and β-isoforms, and of β- and γ- homologues are inactive but the activity of taipoxin is fully replicated by the equimolar combination of α- and γ-isoforms. The molecular basis of the synergistic activity is not characterized.

Crotoxin, a Type2a sPLA_2_ from the venom of the South American rattlesnake *Crotalus durissus terrificus* (see Figure 1), and other related species, is a non-covalently linked complex of crotoxin B (CB) and crotoxin A (CA). CB is a basic single chain of 122 residues. It is hydrolytically active and mildly neuro-myotoxic. CA is a catalytically inactive, non-toxic polypeptide comprising three chains, α, β, and γ, stabilized by five disulphide bridges and derived from the post-translational modification of a “parent” acidic polypeptide of 122 residues. The two sub-units of crotoxin form a stable complex as the result of the formation of polar bonds between Trp residues at homology positions 31 and 70 of CB and Asp 99 and 89 on the β-chain of CA [12]; they can be reversibly dissociated in the presence of urea or in an acidic medium (pH < 2). Although CA is neither hydrolytically active nor neuro-myotoxic the combination of the two subunits results in a decrease in the hydrolytic activity of CB because the substrate binding site on the CB sub-unit is occluded in the presence of the CA sub-unit. The presence of CA also reduces the low-affinity binding of CB to biological substrates and enhances high affinity binding to its primary target on neuronal membranes [12]. This property gave rise to the concept that CA acted as a chaperone to CB. Once the CB sub-unit has bound to its target site the complex dissociates and CA is released.

As with other venom-derived sPLA_2_s, an individual sample of crotoxin containing venom may contain a number of sPLA_2_ isoforms. Four isoforms of both CA and CB may co-exist. These are conventionally referred to as CA1, CA2, CA3 and CA4, and CBa2, CBb, CBc and CBd respectively. Sixteen CA/CB complexes could theoretically exist, all of which have been formally isolated and fully characterised. It is clear that the toxicity and stability of crotoxin depends on the specific CB isoform involved in the formation of the complex. Complexes containing CBb, CBc or CBd are both more toxic and more stable than those containing CBa2 [12]. CB isoforms are also capable of forming heterodimers [13]. This dimerisation is inhibited by CA but it has been speculated that, *in vivo*, the binding of the crotoxin complex, and the release of the CA sub-unit might allow the formation of the CB dimer and the full expression of neurotoxicity [13]. 

β-Bungarotoxin is a major toxin in the venom of kraits that was first isolated from the venom of *Bungarus multicinctus*. The toxin comprises two covalently linked sub-units, Chains A and B. Chain A is a Group 1 sPLA_2_ and chain B is a small polypeptide homologous with the Kunitz-type proteinase inhibitors from the mammalian pancreas. 

**Figure 3:**
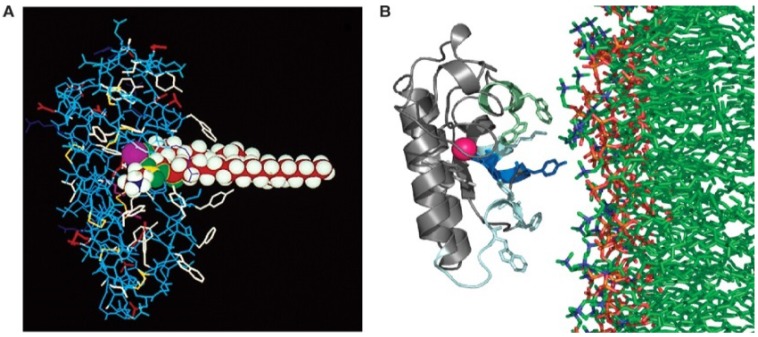
(**A**). A group 1A phospholipase A_2_ with phospholipid substrate modeled in the active site. The active site residues His-48 and Asp-99 and the bound Ca^2+^ is shown in purple. Ca^2+^ is bound by Asp-49 as well as the carbonyl oxygens of Tyr-28, Gly-30 and Gly-32. Aromatic residues are shown in white; (**B**). Model of the lipid binding of the group 1A PLA_2_ is shown with residues on the interfacial binding surface Tyr-3, Trp-19, Trp-61 and Phe-64 shown in stick form. From Burke and Dennis 2008 [1].

Despite these differences in the organisation of toxic sPLA_2_ the mechanism of hydrolysis appears to be identical. The catalytic site of the enzyme is located within a cleft surrounded by a ring of hydrophobic residues [2,14]. The substrate is bound within this cleft, thus shielded from the bulk environment, the Ca^2+^ stabilising the positioning of the substrate within the active site (Figure 3).

## 3. Cytosolic PLA_2_s

Cytosolic PLA_2_s are found in all but a few cell types in the mammalian body. The cPLA_2_s are much larger than sPLA_2_s (cPLA_2_ mass: 40,000–100,000 Da cf sPLA_2_ mass: 13,000–15,000 Da). Like sPLA_2_s, the cPLA_2_s are Ca^2+^-activated but, unlike the sPLA_2_s, calcium is not required for catalytic activation but acts via an N-terminal domain involving Asp-37, Asp-43, Asp-93 and Glu-100 to promote the binding of the enzyme to lipid membranes. In resting cells, at [Ca^2+^]_i_ of 50–100 nM, the enzyme is soluble and largely inactive. An increase in [Ca^2+^]_i_ to 0.3–1.0 µM results in the translocation of the enzyme to its target sites, including the inner leaf of the plasma membrane, nuclear membranes, and the membranes of mitochondria, synaptic vesicles and endoplasmic reticulum. Lipids containing arachidonic acid at the sn-2 position are preferentially hydrolysed by cPLA_2_s leading to the release of arachidonic acid, leaving the relevant lysophospholipid in place [1,3,4,5]. Under stable conditions the production of lysophospholipids is controlled by active re-acylation of lysophospholipids and their re-incorporation as phospholipids into cell membranes. Arachidonic acid is a major precursor for the pro-inflammatory mediators leukotreines, thromboxanes and prostaglandins [2]. 

## 4. On the Evolution of Venom and Its Individual Toxins

Snake venoms are highly complex mixtures of numerous toxic components including phospholipases, proteases and compounds that cause, for example, abnormalities of coagulation, haemorrhage, inflammation, neuromuscular weakness and pain. Snake venoms appear to have evolved in early snakes before evolutionary divergence and the appearance of the advanced snakes. The first stage of this process appears to have been the recruitment of a number of functionally significant proteins into a snake venom proteome and their eventual inclusion, as toxins, in a venom gland associated with an effective delivery system, the proteroglyphic fang. Subsequent to this stage was the secondary independent recruitment of other specific proteins, including sPLA_2_s, into the venom glands of viperidae and elapidae [15]. Snake venoms have been thought to have a several roles: defence/offense against hostile predators; the subjugation of prey items; the initiation of digestion prior to complete ingestion; the ability to target novel prey items when preferred the prey is unavailable. The importance of venoms in the delivery of such basic biological activities means that venom proteins are under intense selective pressure. This has resulted in a form of accelerated evolution involving gene duplication and modification, and the structural and functional diversification of the toxin involved. These processes underpin the evolutionary development of individual toxins and the complex cocktail of individual toxins that together comprise multifunctional, toxic venoms [16,17,18,19].

Snakes need to defend themselves against potential predators. Does this need have a role in driving the evolution of modern venoms? The dominant predators of snakes are birds; minor predators are other snakes and small mammals such as mongooses, opossums and wild cats [20,21,22]. Defensive strategies in snakes are primarily behavioural-fleeing for cover or immobilisation. If escape is not possible or the snake is molested the behaviour becomes actively threatening with open mouth, lifting the body into strike position, hissing, lunging, vibrating the tail. Aggressive biting and envenoming is relatively uncommon unless the snake is molested and unable to retreat [20] although there is anecdotal evidence that males are more aggressive when with females and females are more aggressive when they are isolated or with young [23]. Envenoming of predators is often ineffective because so many predators are either covered with fur or feathers and/or have evolved either circulating serum proteins that inhibit the activity of major venom toxins, or the development of junctional AChR that are resistant to the binding of post-synaptically active neurotoxins [22,24,25,26]. It would be of interest to know whether predators behave differently when confronting a venomous or a non-venomous snake—or indeed whether they learn to distinguish between them at all. It seems unlikely that defence is a major factor involved in the evolution of venoms and toxins. 

The ubiquitous presence of proteases and phospholipases, alongside neuro- and haemotoxins in snake venoms have led to the suggestion that neurotoxins immobilize prey items, haemotoxins inhibit coagulation and enable the continuing circulation of incoagulable blood. The combination enables proteases and phospholipases to circulate and initiate the breakdown of cells and tissues before the complete ingestion of the envenomed prey [27,28,29]. The involvement of venom in the immobilisation of prey may be significant if the prey item is potentially able to damage the snake but reports that consumed prey can remain alive and mobile even after ingestion are numerous [30,31]: moreover, numerous venomous species use constriction as well as or instead of envenomation as a means of immobilising prey [20]. 

Formal studies of the role of envenomation in the initiation of digestion are few. Thomas and Pough [32] examined digestion of mice loaded with “rattlesnake venom” fed live to non-venomous snakes and reported accelerated digestion only at 15 °C. McCue [33] examined metabolic expenditure, passage time and assimilation efficiency in *Crotalus atrox* fed freshly killed mice inoculated with *C*. *atrox* venom and found no improvement in digestive efficiency. Until similar work has been made using other venoms, natural prey items and natural forms of envenomation it must be concluded that there is no credible evidence that a digestive function for venom is a major factor in the evolution of snake venoms. 

Is availability of food a major force in the accelerated evolution of venoms and toxins? The diet of a snake can vary considerably over a season and it is well established that, in larger snakes, the diet changes from one that primarily comprises ectotherms (typically amphibians, small reptiles) to one that includes endotherms (typically mammals) as the snakes grow [34,35]: in some species the change in diet appears to be related to the onset of sexual maturity [20]. Eating behaviour may also be modified according to the type of prey. For example, adult tiger snakes, *Notechis scutatus*, bite and hold amphibians but bite and release potentially dangerous rodents [36]. It is also well established that venom composition of a given species can vary according to geographical region [37,38,39]. It is generally supposed that this reflects differences in prey items in different geographical regions and that this intra-species variation has evolved to enable the snake to utilise different prey items according to availability. If this were to involve the consumption of ectotherms in one region and endotherms in another it might be expected that the greater difficulty of tackling and digesting the larger, fur covered mammal than a frog might require the inoculation of a more toxic venom. If those populations of snakes were isolated one might also expect the composition of their respective venoms to be determined ultimately by heritable genes which could inhibit their ability to adjust to environmentally-driven changes in prey items [27]. A significant problem in determining the precise relationship between venom evolution and composition, and the availability of prey is that the toxicity of venoms and toxins is almost always related to toxicity towards laboratory mice or rats rather than actual prey items. There is a general lack of detailed knowledge of those local prey items and the way snakes choose which potential prey items to consume. Is it acquired and determined according to the first experience of feeding or by the relative abundance of one item over another or is it inherited?

Resolving some of these questions on the factors involved in the evolution and composition of toxins and crude venoms is important for taxonomic studies but is of critical importance in clinical medicine because intra-species variations in the composition of venoms can affect both the initial diagnosis of biting species and clinical prognosis. Warrell, [40] has documented the clinical problems associated with envenoming bites by a number of snakes with very large ranges. For example, envenoming by *Daboia* (previously *Vipera*) *russelli*, a viperid snake with a very extensive but discontinuous range across South Asia, is associated with a general increase in capillary permeability and bleeding into the pituitary in Burma, but in Sri Lanka and South India, rhabdomyolysis is common. *Crotalus durissus* inhabits a wide range in Brazil and Argentina. The venom of the some populations of the snake contain the toxin crotamine. In other populations crotamine is absent. Envenoming bites by the snake cause severe myotoxicity and neurotoxicity if the venoms contain crotamine and minimal neuro-myotoxicity in those where crotamine is absent. Envenoming by the cobra *Naja kaouthia* causes severe neurotoxicity and modest local necrosis in the Philippines and Bangladesh but in Malaysia local necrosis is the dominant feature and clinically severe neurotoxicity is uncommon. 

These intra-species variations in the composition of venom are also responsible significant problems with antivenoms when the venoms used to prepare an antivenom have been collected from snakes from only one part of its range but is to be used elsewhere. Ideally, the venoms should be collected from snakes of all ages, both sexes and from all parts of their natural range if an antivenom is being designed for use across its range [40]. 

## 5. Snake Bites and Associated Neuro- and Myotoxicity in Humans

**Figure 4:**
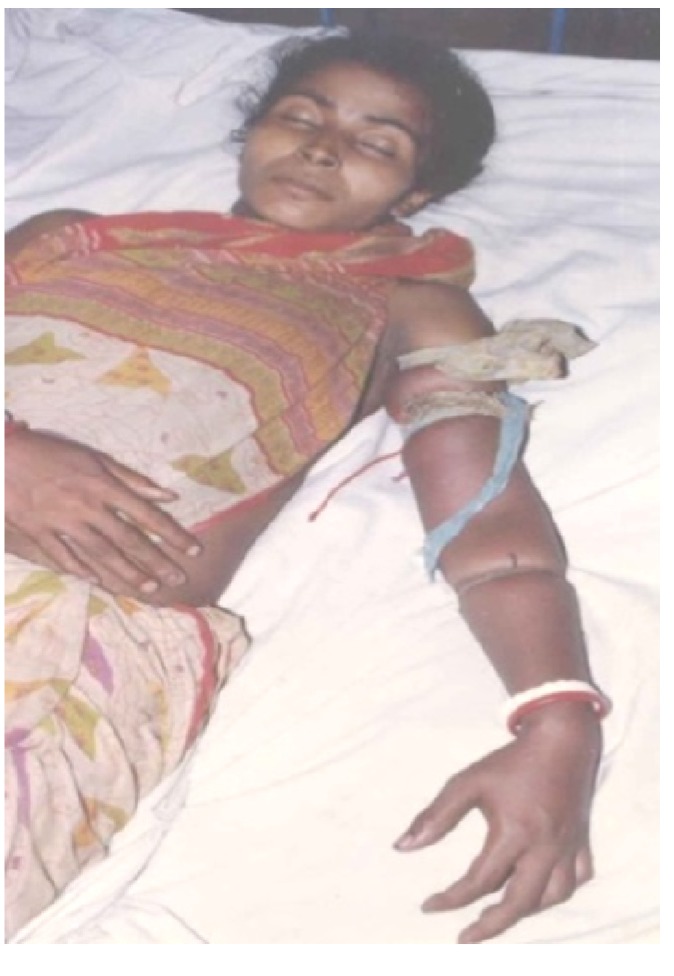
Victim of an envenoming bite by an unidentified snake on admission at a tertiary referral hospital in Chittagong, Bangladesh. Note multiple tight ligatures applied to the arm.

Snake bite is a significant public health problem in many parts of the world, including Southern Europe, North America and Australasia, but is a major problem in rural sub-Saharan Africa, South East Asia and South America. Effective treatment requires the accurate identification of the biting snake. Patients rarely bring the offending animal to the clinic. In many cases the animal is not seen and even if it is seen patients can often neither identify nor accurately describe it. Treatment, therefore, often depends on the clinical attendant being able to adopt a syndromic approach to identification and then choose the most appropriate treatment [41]. An additional problem for the clinician is that serious clinical signs of envenoming may develop very slowly. This may lead to the erroneous conclusion that the bite is by a non-venomous snake, or a bite by a venomous snake without the inoculation of venom (a defensive “dry bite”) or with the inoculation of a clinically insignificant amount of venom. For this reason there is a general recommendation that all patients with a suspected snake bite should be kept under observation in a clinical facility for 12–24 h before discharge. Finally, in poor rural areas, it can take many hours before a bitten patient reaches a competent clinical facility. Most of those patients will have received first aid at the time of the bite and will probably have also been seen by a local healer before being referred onwards. Most patients will have had ligatures applied proximal to the bite site (Figure 4). 

**Figure 5:**
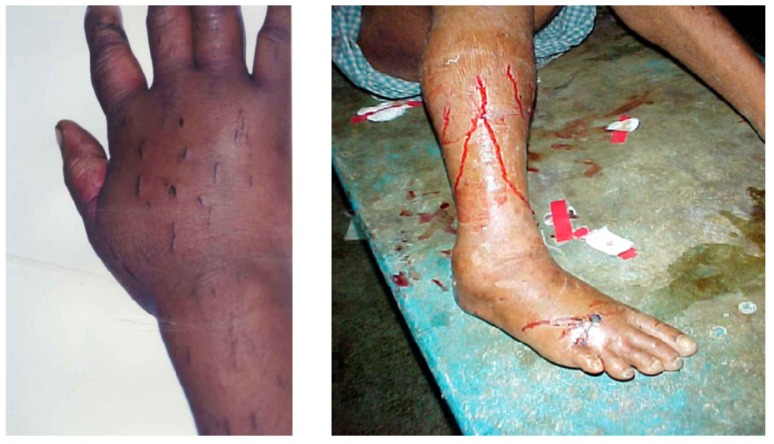
Incisions applied to the hand and lower leg respectively in two victims of envenoming bites by unidentified snakes in Chittagong, Bangladesh.

Many will have also been given noxious, emetic, infusions to drink and herbs, mud or stones may have been applied to the wound. Incisions are often made over and around the site of the bite and elsewhere on the bitten limb in an attempt to release the venom (Figure 5). 

By introducing the possibility of severe bleeding, infection, ischaemia, pain and vomiting local healers can make both the treatment and management of the acutely ill patient, and the analysis of outcomes, more complex than is generally assumed [41,42,43]. 

**Figure 6:**
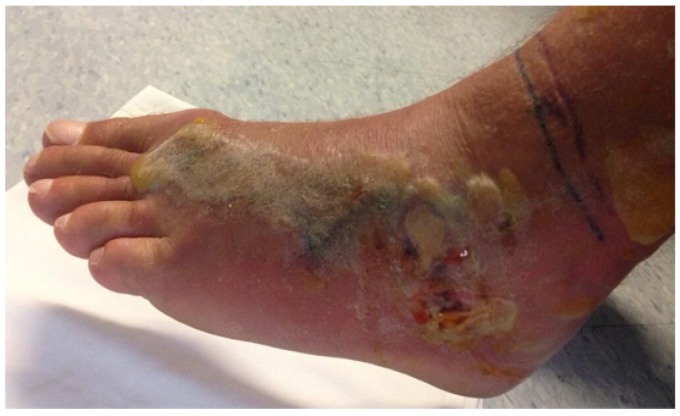
Localised necrosis following an envenoming bite to the foot by the viperid Fer de Lance, (*Bothrops asper*).

Venoms are complex mixtures of biologically active small molecules and polypeptides. The individual components of the venom have diverse pharmacological targets in a variety of cells and tissues. As a result, on presentation to a clinical attendant, the victim of a snake bite will present with an array of non-specific and specific signs and symptoms. Anxiety is common. Headache, local pain and swelling, vomiting, nausea and fainting are all common non-specific signs. Lymphatic pain and swelling is often associated with envenoming bites and arises because the inoculated components of the venom enter the lymphatic system prior to entering the circulation. Systemic envenoming typically presents with either: major haemotoxic reactions resulting from the activity of haemorrhagins, procoagulants, anticoagulants, platelet inhibitors and activators and haemolytics; neuromuscular weakness as a result of the presence in the venom of toxins that block AChRs at the neuromuscular junction; or neuro-myotoxicity resulting from toxins that initiate neuro- and/or myo-degeneration [44,45]. Local bruising or blistering and soft tissue necrosis may arise as a result of cytotoxins in the venom of many snakes, including cobras and most viperids and crotalids (Figure 6).

**Figure 7:**
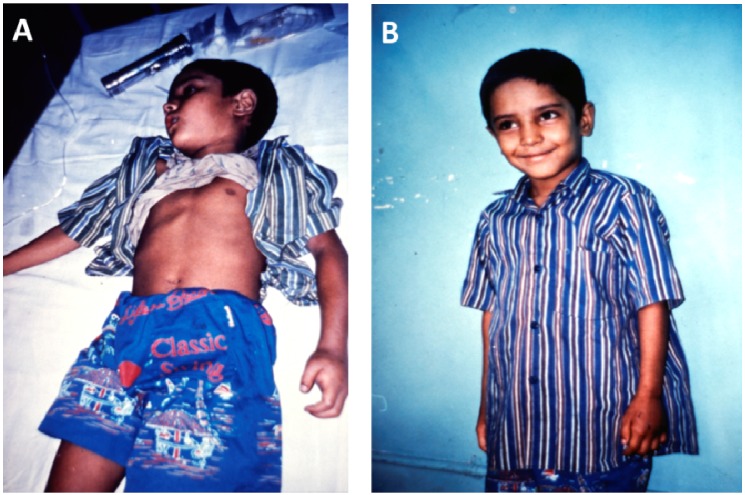
(**A**) Young boy with severe neurotoxic signs following a bite by a cobra (species unknown) in Bangladesh; (**B**) Full recovery 24 h later following treatment with antivenom.

The most important neurological consequences of envenoming bites are those that result from bites by the elapids of Africa, Australia and Papua New Guinea and SE Asia, and the elapids and some viperids and crotalids of the Americas. The venoms of elapids, and some viperids and colubrids, contain post-synaptically active neurotoxins that bind to and block the junctional ACh receptors [46]. The result is a delay of up to 30 min before the onset of ptosis, exophthalmoplegia, difficulty in swallowing and speaking, difficulty in opening and closing the mouth and a progressive, generalised, descending neuromuscular weakness. These toxins can cause a fatal neuromuscular paralysis but the paralysis can usually be reversed by treatment with appropriate antivenoms or anticholinesterases (Figure 7) [47,48,49]. 

Myotoxicity, often associated with general, localised cytotoxicity is a feature of bites by a number of viperids and crotalids whose venoms often contain small polypeptides known collectively as myotoxins. 

**Figure 8:**
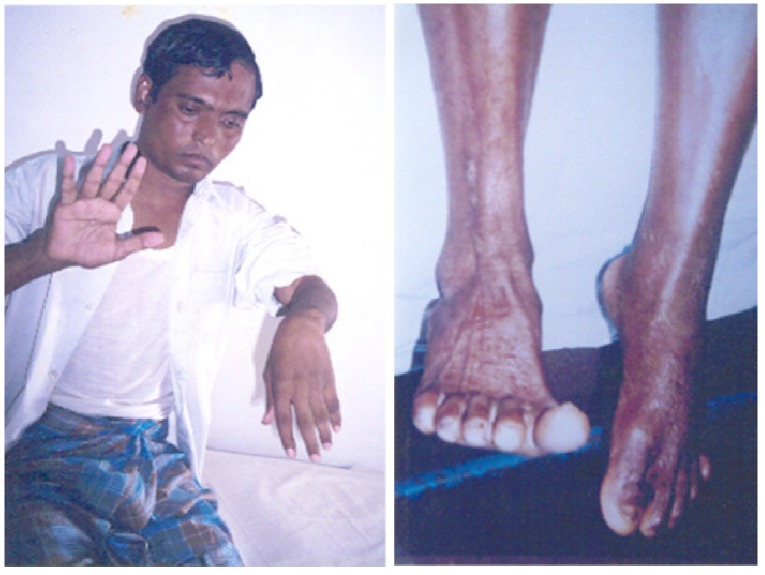
Wrist drop and foot drop, respectively, many months after the apparently successful treatment of victims of neurotoxic snake bites in Chittagong, Bangladesh.

A more severe failure of neuromuscular transmission follows envenoming bites by snakes whose venoms are rich in sPLA_2_s—typically the elapids and sea-snakes of S.E. Asia and Australia/Papua New Guinea some elapids, viperids and crotalids of the Americas and Europe. Envenoming bites by these snakes can give rise to prolonged neuromuscular paralysis that is resistant to treatment with either antivenoms or anticholinesterases [50,51,52,53]. For example, a major Sri Lankan study of more than 200 patients bitten by common kraits (*Bungarus caeruleus*), whose venoms are particularly rich in toxic PLA_2_s, reported ptosis, exophthalmoplegia, dysphagia, dysphonia and neuromuscular weakness as common signs of envenoming. Half of all patients in this study had a tidal volume below 200 mL (normal tidal volume in an adult male is approximately 500 mL), neck flexor power of less than 3 (on a range of 1–5 where 5 is no discernible weakness) and required assisted ventilation. Severe neuromuscular weakness lasted for between 12 h and 29 days with a median of between 2 and 4 days before recovery began. Assisted ventilation may be required for many days before spontaneous respiration begins. Thereafter both generalised neuromuscular function and power is restored rapidly. A clinical neurophysiological study of patients bitten by taipans in Papua New Guinea showed that following an envenoming bite the development of life-threatening neuromuscular weakness was associated with a rapid fall in the amplitude of the compound muscle action potential. Between 3 and 4 days after the bite the amplitude of the compound muscle action potential and grip strength began to increase to become essentially normal by 10–20 days [51].Connolly *et al*. also reported that, during recovery, single fibre EMG showed extensive jitter and blocking, features consistent with axonal regeneration and the re-innervation of denervated muscle fibres [52]. Whether regeneration is sustainable in the long term is difficult to judge. Follow up studies are exceptionally rare in those regions where neurotoxic snake bite is common. It is, however not uncommon for patients to complain of residual problems like wrist and foot drop [53], (Figure 8). 

It is usual for these problems to be considered the result ischaemia following the use of over-tight ligatures but it remains possible that the regenerated axons and their parent neurons are “weak” and prone to undergo delayed, secondary degeneration (see Section 6 below). 

**Figure 9:**
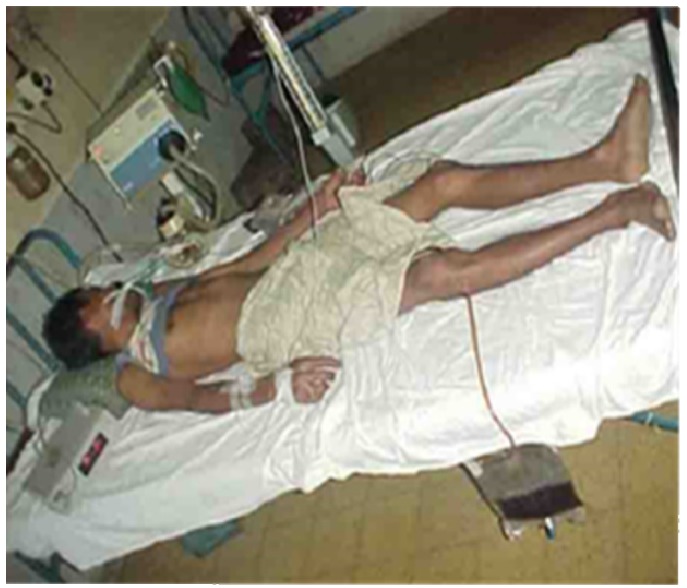
Severe neurotoxicity and rhabdomyolysis (note the black urine) following an envenoming bite by a greater black krait, *Bungarus niger* in Bangladesh. The patient did not recover.

**Figure 10:**
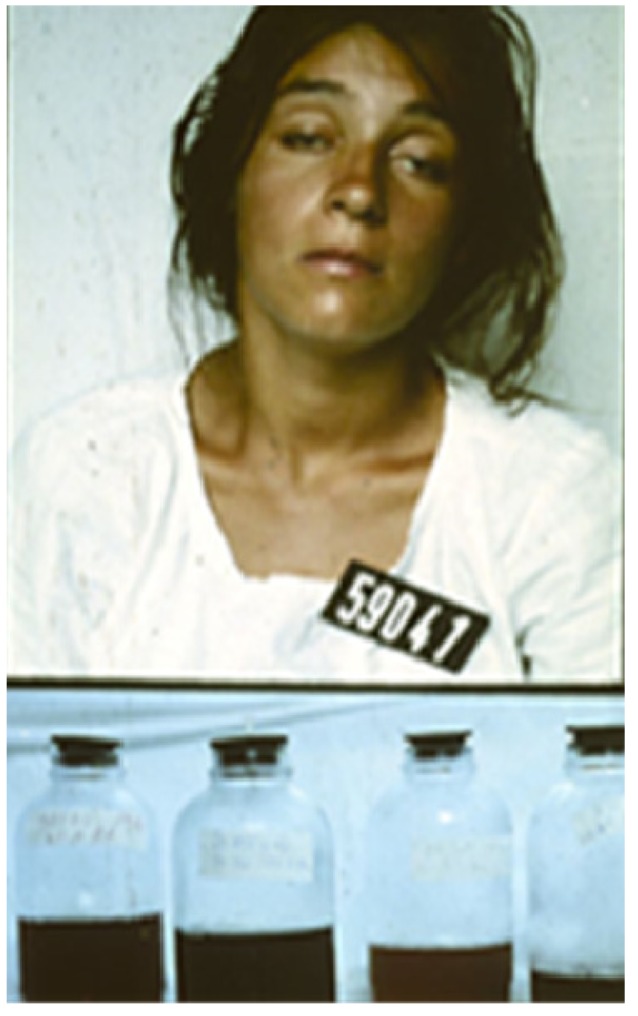
Ptosis and rhabdomyolysis (note the black urine) following an envenoming bite by South American rattlesnake (*Crotalus durissus*) in Brazil.

Reid, working in Penang, colonial Malaya, during the 1950s and 1960s, was the first to describe in detail the severe, life threatening muscle degeneration (rhabdomyolysis) associated with myalgia, hyperkalaemia and myoglobinuria following envenoming bites by sea snakes [54,55,56]. Reid’s work excited little immediate attention and myotoxicity was almost completely ignored as a major clinical problem in snake bite until reports of myalgia associated with cases of acute renal failure following bites by a number of Australian elapids began to appear [57,58,59,60,61,62]. Severe myotoxicity, often associated with neurotoxicity, is now more widely recognised as a common problem following bites by many elapids and viperids (Figure 9 and Figure 10).

It remains probable that many cases are missed because few rural clinics are able to recognise the significance of generalised muscle pain or to record hyperkalaemia, elevated serum creatine kinase or myoglobinuria. Thus stained urine is frequently interpreted as haemoglobinuria without a consideration of myoglobinuria [63]. Similarly, a positive dipstick test for the presence of heme in the urine (a procedure sometimes used in rural clinics) does not allow the distinction between haemoglobin and myoglobin and is usually interpreted as blood in the urine. The accurate recognition of myotoxicity is important because of the potential for myoglobinuria to cause acute renal failure [62,63]. A practical example of the problem of the recognition of myotoxicity relates to envenoming bites by kraits (genus *Bungarus*). The kraits form a distinctive group of thirteen species of SE Asian elapids. It has long been assumed that envenoming bites by kraits do not cause myotoxicity. Laboratory studies of the myotoxic potential of the venoms of kraits have shown, however, that the venoms of *B*. *candidus* and *B*. *fasciatus* cause dose-dependent muscle necrosis in rats [64]. It is now clear that envenoming bites by a number of species of krait, including *B*. *caeruleus*, *B*. *candidus*, *B*. *niger* and *B*. *multicinctus* (in Viet Nam) can cause severe myotoxicity in patients [65,66,67,68,69]. 

Detailed studies of the pathology of skeletal muscle and peripheral nerve in human victims of snake bite are difficult to make, especially in the rural areas of Africa, SE Asia and South and Central America where envenoming bites typically occur. Apart from the absence of appropriate clinical and laboratory facilities such studies on critically ill patients, many of whom also have a venom induced coagulopathy, would be difficult to justify on ethical grounds. Clinical observation may allow an educated guess as to underlying pathophysiology but do not allow a definitive understanding of the biological basis of the severe paralysis seen in so many patients. Experimental studies, made *in vivo* on intact animals and *in vitro* using isolated cells and tissues, on the pharmacology and physiology of snake venoms and their component toxins have been particularly important; they have contributed greatly to our understanding of the biological basis of both neuro- and myotoxicity and the cellular mechanisms involved. 

## 6. Experimental Studies of the Neuro- and Myotoxicity of Venom-Derived Phospholipases

There are several caveats to consider when extrapolating findings generated from studies made using experimental animals and non-human tissues to the syndromes expressed in envenomed humans. Among the most important are the following:

- The toxicity of a venom or toxin often varies greatly between species. Thus, a venom/toxin might be very toxic to a laboratory animal but of little significance to a human [70].
- Even if the venom or toxin is potentially dangerous to a human, the snake may be incapable of inoculating sufficient venom to be dangerous to a human.
- Bitten humans are inoculated with complete venom rather than individual purified venom constituents. As a result, the syndrome associated with envenoming arises from the activity of a number of biologically active constituents. Thus care needs to be exercised when assessing the contribution a single toxin might make to the overall syndrome expressed in a human subject [71].
- The length of the fangs of elapids, rarely exceeds five mm. The inoculation of venom by an elapid into a human subject is, therefore, rarely directly intramuscular or intravenous and is never intraperitoneal [20].
- Although circulating concentrations of venom in patients can be directly measured using, for example, immunoassay techniques, there are limited data available in the literature for all but a few species of biting snake. Where such measurements have been made, the circulating concentration of venom is typically between 30 and 2000 ng/mL [72,73]. Alternatively, where both the amount of venom in the venom glands and the body weight of the typical human subject are known an approximate dose of venom received by a bitten subject can be calculated [68]. Rarely are such calculations made in laboratory-based studies and so the relevance of the findings to the human situation is often difficult to assess.
- The majority of venom proteins do not cross the blood brain barrier. This raises the question of whether the findings of studies involving the direct inoculation of venoms/toxins into the CNS, or their direct application to brain slices or cultures of neuronal cells, are of any direct relevance to the clinical aspects of peripheral neurotoxicology.

These issues need to be more widely understood when experimental work is being designed and results interpreted, especially when the objective is to understand the clinical signs and symptoms of envenoming. 

## 7. The Neurotoxicity of Venom-Derived sPLA_2_s

The peripheral nervous system is particularly susceptible to attack by neurotoxins because the terminal parts of the motor axons and the terminal boutons are not protected by either a blood-axon barrier or a perineurium. The nerve terminals are also a long distance from the parent cell body and, accordingly, rely on an extremely efficient system of both anterograde and retrograde transport for their maintenance. The neurotoxic sPLA_2_s are presynaptically active, targeting the motor nerve terminal and the terminal part of the motor axon. They do not bind to or block junctional ACh receptors (although at high concentrations they may stablilise the ACh receptor in its desensitised state). Most neurotoxic PLA_2_s are also myotoxic (see below). The major exception to this generalisation is β-Bungarotoxin, the major presynaptically active neurotoxin in the venom of *Bungarus multicinctus*, the banded krait of Taiwan [74]. 

**Figure 11:**
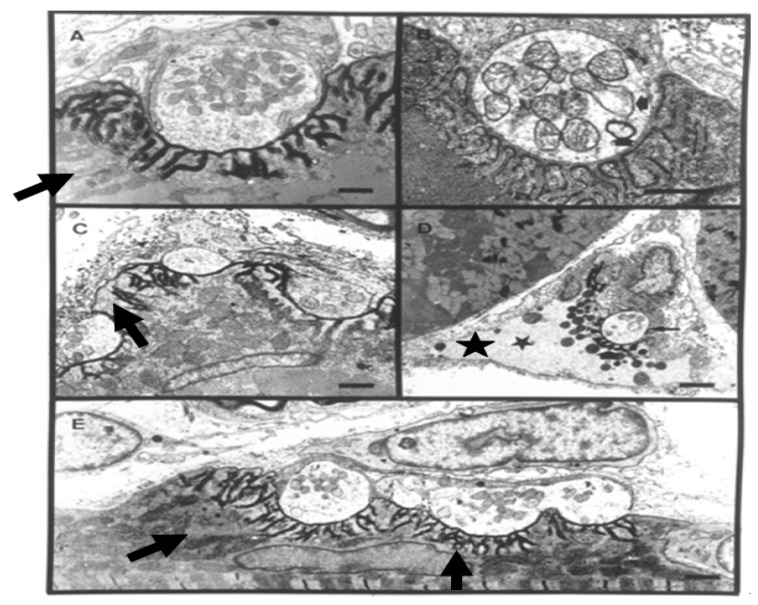
TEM Images of motor nerve terminal boutons on muscle fibres of the rat 12–24 h after the inoculation of notexin, a PLA_2_ toxin from the venom of the Australian tiger snake, *Notechis scutatus*. (**A**) Control bouton on a muscle fibre not exposed to any toxin. Note the folds of the postsynaptic membrane (**Arrows**); (**B**–**E**) Note the widespread loss of synaptic vesicles from the boutons and the swollen mitochondria (**small arrows**). Note also the well preserved junction folds of the neuromuscular junctions (**large arrows** in **C**). Combined damage to both bouton and muscle fibre is shown in **D**: a **star** marks the collapsed muscle fibre but note the preservation of the junctional folds at the neuromuscular junction.

The pharmacology of the neurotoxic sPLA_2_s is complex and confusing. Isolated skeletal neuromuscular preparations exposed to the toxins exhibit a lag phase of 3–10 min, during which the toxins can be removed by washing or inactivated by specific anti-toxins or relevant anti-venoms [75,76]. This lag phase is generally considered to reflect the time taken for the toxin to bind irreversibly to its substrate or become internalised by the target cell. Following the “binding stage” there follows the development of a poorly understood triphasic response that comprises a first phase of reduced spontaneous and evoked transmitter release followed by a second phase of increased release and a final phase of progressively declining transmitter release ending in a complete failure of transmission. This triphasic sequence of events is highly variable and depends on the toxin involved, the species from which the neuromuscular preparation was obtained, the rate and pattern of indirect stimulation, temperature and the relative concentrations of Ca^2+^ and Mg^2+^ [11,77,78]. For the clinical toxicologist the crucial problem is the ultimate, prolonged, treatment-resistant failure of neuromuscular transmission. This feature is often described as transmitter blockade but it is now clear that the problem is more likely caused by the depletion of synaptic vesicles in the terminal bouton and, possibly, by a reduction in the recycling of synaptic vesicles following exocytosis (Figure 11).

**Figure 12:**
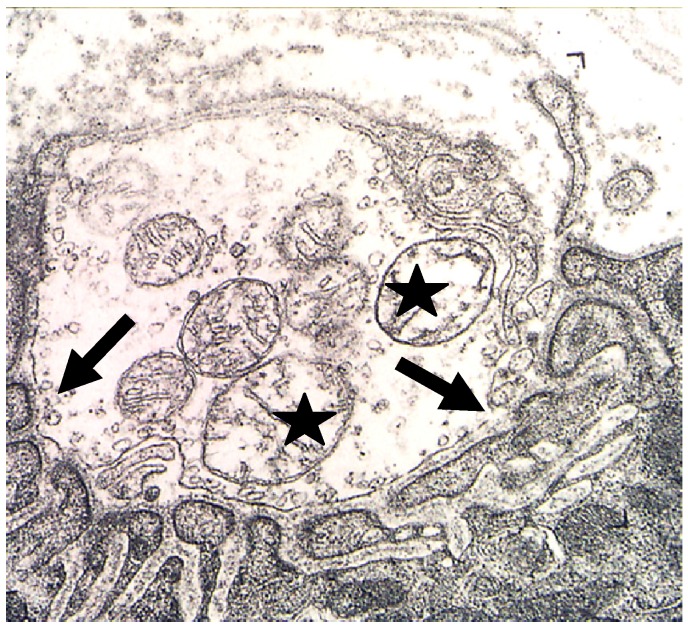
A terminal bouton in advanced stages of degeneration. Note the lesions in the plasma membrane (**arrows**) and the damaged mitochondria (**stars**).

**Figure 13:**
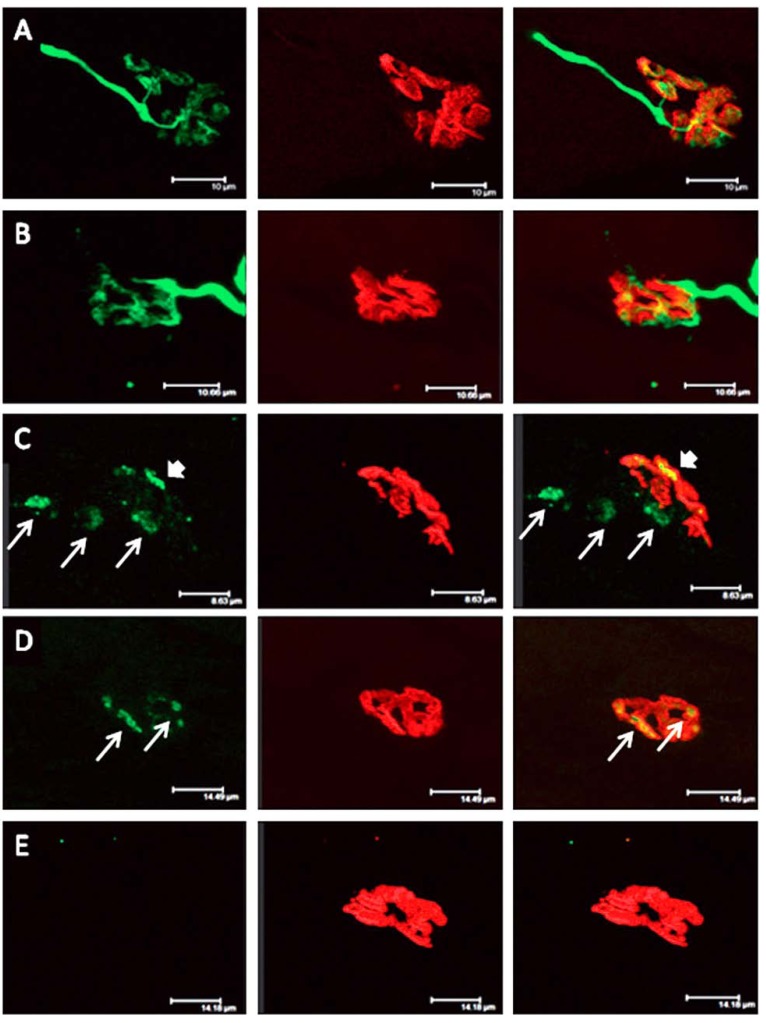
Longitudinal sections of rat soleus muscles 24 h after the inoculation, *in vivo*, of the venom of the Greater black krait, *Bungarus niger*. Sections were labelled with TRITC-conjugated α-Bungarotoxin to label junctional ACh receptors (**red**) and FITC conjugated Ab to neurofilament protein to label motor axons (**green**). (**A**) control image; (**B**–**E**) Progressive breakdown of the terminal innervation at the neuromuscular junction. Note the preservation of the junctional ACh receptors (From Faiz *et al*. 2010 [68]). Reproduced with permission from Publisher.

Ultimately the nerve terminal and the distal parts of the motor axon degenerate (Figure 12 and Figure 13) [79,80,81,82,83]. 

**Figure 14:**
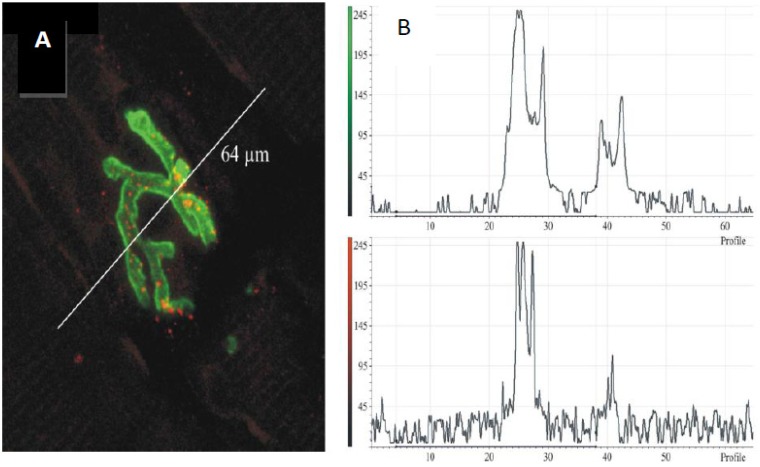
(**A**) Longitudinal section of murine muscle labelled with ammodytoxin A, an sPLA_2_ from the venom of the long-nosed viper, *Vipera ammodytes*, conjugated with Alexa^546^ (**red**) and counter-labelled with FITC-conjugated α-Bungarotoxin to label junctional ACh receptors (**green**) (**B**) a laser scan of red and green channels to demonstrate localisation of sPLA_2_ to the neuromuscular junction. (From Logonder *et al*. 2008 [85]). Reproduced with permission from the Publisher.

The biological basis of synaptic vesicle depletion and the degeneration of the nerve terminal and terminal motor axon has been the subject of intensive recent study. The toxins do not influence the organisation or functional behaviour of the post-junctional ACh receptors. Moreover the basic structure of the post-junctional membrane appears to be resistant to structural damage possibly because of its highly structured system of deep folds stabilised by a specialised cytoskeleton [83]. The target appears to be specifically located at the neuromuscular junction [84] (Figure 14). 

**Figure 15:**
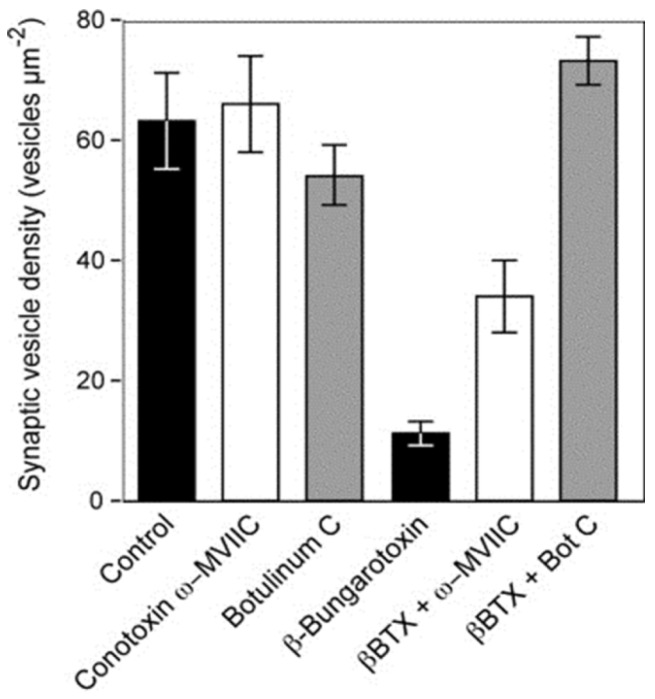
The density of synaptic vesicles in terminal boutons of rat neuromuscular junctions. Vesicle density was unchanged in muscles incubated *in vitro* with either botulinum toxin C or conotoxin ω-MVIIC. Incubation with β-bungarotoxin, an SPLA_2_ toxin from the venom of *Bungarus multicinctus*, caused a significant fall in vesicle density. The fall was largely or completely prevented in muscles pre-treated with either botulinum toxin C or conotoxin ω-MVIIC before exposure to β-bungarotoxin. (From Prasarnpun *et al*. 2004 [76]). Reproduced with permission from the Publisher.

Rigoni *et al*. 2005 and Caccin *et al*. 2006 have shown convincingly that the extracellular presence of lysophospholipids and fatty acids, particularly lysophosphatidylcholine, reproduces in many regards the pathology that follows exposure to the neurotoxic sPLA_2_ s [78,85]. Thus it can be reasonably considered that the primary event in the initiation of nerve terminal pathology begins with the hydrolysis of the lipids of the outer leaf of the plasma membrane of motor nerve terminals. The resulting instability of the plasma membrane of the nerve terminal is thought to result in depolarisation of the nerve terminal, the entry of Ca^2+^ via activated voltage gated Ca^2+^ channels and an inevitable increase in exocytosis. Some of the best characterized neurotoxic sPLA_2_s bind to, and block pre-junctional voltage gated K^+^ channels [86,87]. In these circumstances nerve terminal depolarization would be expected to last longer thereby reinforcing the elevation of entry of Ca^2+^ because voltage gated Ca^2+^ channels would be expected to remain in an open state for longer. Prasarnpun *et al*. 2004 have shown that depletion of the synaptic vesicles can be largely prevented by exposure to conotoxin ω-MVIIC, which selectively blocks the opening of P/Q type voltage-gated Ca^2+^ channels that populate the mammalian motor nerve terminals, and botulinum toxin C which hydrolyses syntaxin and SNAP-25, thus preventing the formation of SNARE complexes that underpin exocytosis [76] (Figure 15). 

**Figure 16:**
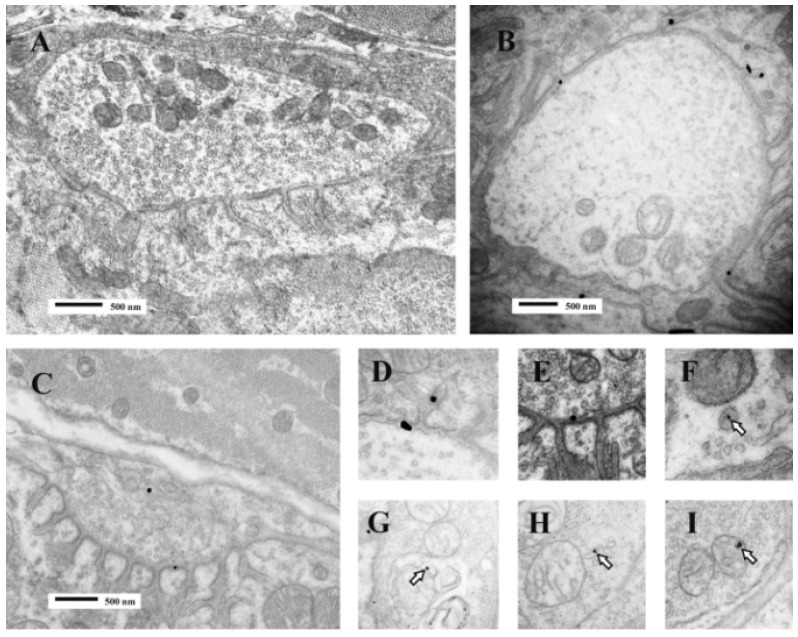
TEM images of terminal boutons on murine muscle fibres previously exposed to a gold-labelled sPLA_2_ from the venom of the horned viper (*Vipera ammodytes ammodytes*). The control bouton (**A**) is not decorated; Bouton **B** shows particles within the synaptic cleft and folds. Bouton **C** shows particles in the synaptic cleft and the bouton itself. Enlarged images (**D**–**I**) show particles associated with vesicle–like structures within the bouton or with mitochondria (**open arrows**). The association between label and vesicle-like structures suggest that uptake might occur during the recycling of synaptic vesicles and endocytosis. (Modified from Logonder *et al*. 2009 [94]). Reproduced with permission from the Publisher.

These data confirm that the entry of Ca^2+^ into the nerve terminal via open voltage-gated Ca^2+^ channels is an important part of the process and that enhanced exocytosis relies on the formation of SNARE complexes as in normal neuromuscular transmission. It is probable that the depletion of synaptic vesicles from the nerve terminal is not simply a reflection of enhanced exocytosis; the increased fluidity of the nerve terminal membrane will also inhibit synaptic vesicle recycling. The entry of Ca^2+^ into the nerve terminal would also explain the activation of the Ca^2+^-activated, intracellular, proteolytic enzymes (calpains), the very rapid degeneration of the mitochondria and the degeneration of the neurofilaments of the terminal parts of the motor axon. Similarly, the elevation of [Ca^2+^]_i_ would result in the activation of cPLA_2_ in the cytosol of the motor nerve terminal. Synaptic vesicles are also likely to be destroyed as they are known to be very vulnerable to exposure to both s- and cPLa_2_ enzymes and the products of lipid hydrolysis [88,89,90,91]. There is also evidence that neurotoxic sPLA_2_ can enter the nerve terminal [92,93,94]. In many cases, internalised toxin appeared to be closely associated with vesicle-like structures suggesting that uptake might occur recycling of synaptic vesicles during endocytosis (Figure 16). 

Despite the evidence that sPLA_2_s can enter nerve terminals, at least under experimental conditions, there is an ongoing dabate about the true biological significance of the phenomenon on the grounds that only a small minority of terminals ever show show any evidence of internalisatiotion of the toxins [92,93,94,95]. 

**Figure 17:**
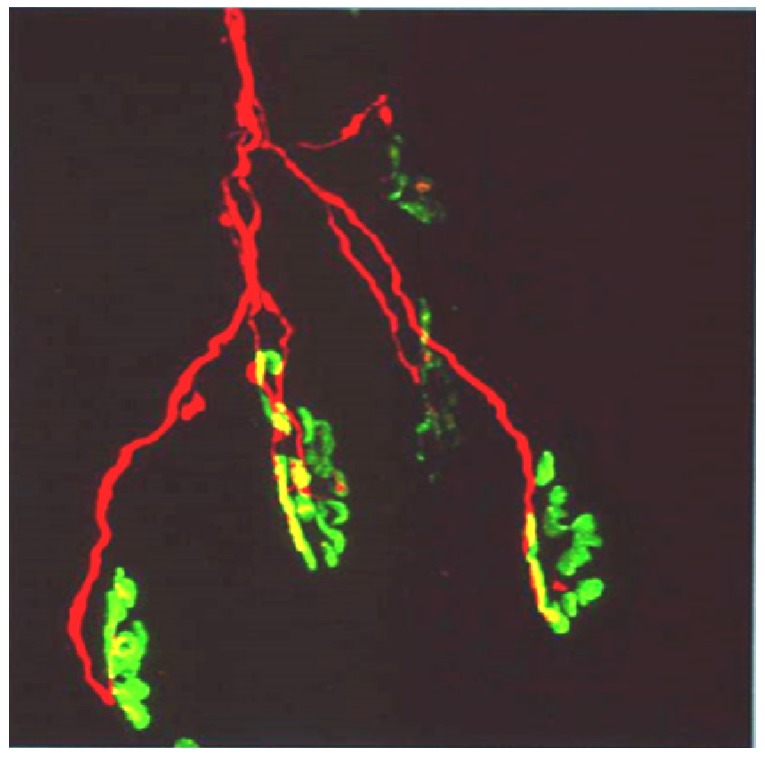
A cluster of six individual end-plates (labelled with FITC-conjugated α-bungarotoxin) innervated by the clustered intramuscular branching of a single motor axon (labelled with TRITC-conjugated anti-neurofilament Ab.

The regeneration of the peripheral innervation following exposure to the neurotoxic sPLA_2_s begins 3–4 days after the initial exposure to the toxins. At 3 days small, partially differentiated growth cones appear in the synaptic trough. By 4 days spontaneous and evoked transmitter release can be recorded. Quantal contents of the end-plate potentials at this stage are small but the amplitude of the miniature end-plate potential is normal. Axonal sprouting is common in the early stages of regeneration but the sprouts are rapidly withdrawn. By 10–14 days the organisation and function of the individual end plates and motor units appear completely normal with the exception of sometimes extensive collateral re-innervation [96,97] (Figure 17).

Whether the organization of the peripheral nervous system remains structurally and functionally “normal” in the longer term is uncertain. Prasarnpun *et al*. [88] have reported a reduction in the number of myelinated axons in the regenerated soleus nerve trunk 6 months after the injection β-bungarotoxin into the ipsilateral hind limb and the same toxin can induce neuronal cell death in a variety of experimental situations [98,99,100,101]. Clearly there is a need for detailed long term studies of motor neuron health in controlled studies in intact animals if this uncertainty is to be resolved. The problem is not of purely academic interest as the long term consequences of envenomation in human subjects remains similarly unclear (see Section 5).

## 8. The Myotoxicity of Venom-Derived sPLA_2_s

**Figure 18:**
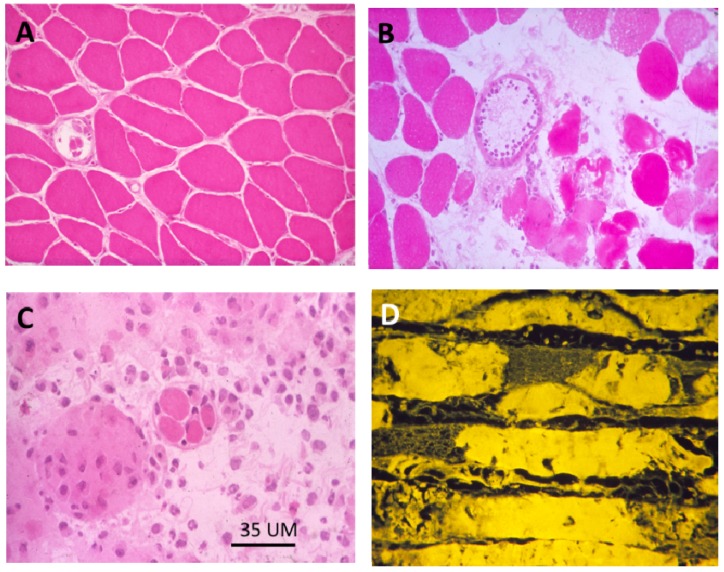
(**A**–**C**) Transverse sections of soleus muscles stained with haematoxylin and eosin (H&E). (**A**) control; (**B**,**C**) Three and 24 h respectively after exposure, *in vivo*, to notexin, an sPLA_2_ from the venom of the Australian tiger snake, *Notechis scutatus*. Note the early inflammatory response and the later degeneration of the muscle fibres; (**D**) Longitudinal section at 24 h stained with procion yellow. This dye is excluded from cells with an intact plasma membrane. Note that it has entered the muscle fibres and stained the congealed, hyper-contracted myofilaments.

Exposure of skeletal muscle to many venom-derived sPLA_2_s causes a severe inflammatory degenerative response. In typical studies, the toxic sPLA_2_ or the relevant native venom is administered either by intramuscular injection or by subcutaneous injection over the muscle to be studied. The first signs of inflammation and myotoxicity, appearing less than an hour after inoculation, are local swelling, the favouring of the inoculated limb and the loss of the toe-extension reflex. Affected muscle fibres are rapidly depolarised following exposure to the toxic sPLA_2_s, membrane potentials falling from between −75 and −80 mV to −5 mV within 6hrs. Histopathology, electron microscopy and gamma-scan imaging of ^99m^Tc-methylene diphosphonate labelling reveals that the swelling is the result of oedema associated with a major inflammatory response and that the degeneration of the muscle fibre is complete by 24 h [102,103,104,105,106,107] (Figure 18).

**Figure 19:**
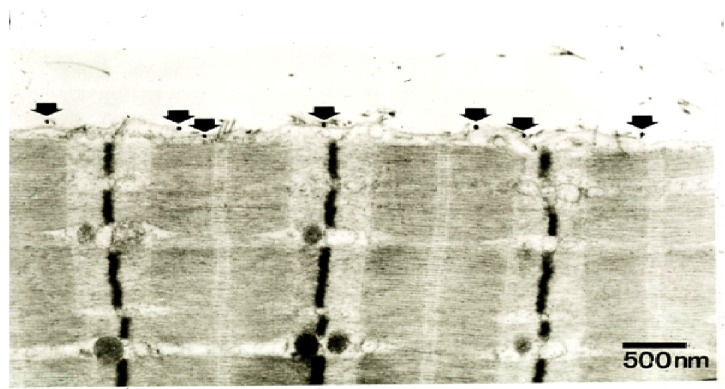
TEM of a longitudinal section of a rat soleus muscle fibre labelled with a gold-conjugated Ab against notexin, an sPLA_2_ from the venom of the Australian tiger snake, *Notechis scutatus*, three hrs after exposure *in vivo* to the toxin. Arrows indicate individual silver-enhanced gold particles. (From Dixon and Harris 1996 [109]). Reproduced with permission from the Publisher.

It is well established that exposure of skeletal muscle to myotoxic PLA_2_ in the presence of Ca^2+^ leads to the hydrolysis of phosphatidyl choline and phosphatidyl ethanolamine, the major constituents of the plasma membranes of excitable cells [88] and that one result is the loss of cytosolic proteins and the uptake of procion dyes and similar agents usually excluded by the intact plasma membrane. The results of experiments of this kind suggested that, *in vivo*, the cell surface is the primary target for the myotoxic PLA_2_s but they were not definitive. More direct evidence of the importance of the plasma membrane was obtained by Brenes *et al*. 1987 who used immunocytochemistry to demonstrate that the binding of a non-hydrolytic myotoxin from the venom of *Bothrops asper* was to the plasma membrane of skeletal muscle fibres, and by Dixon and Harris 1996 who used immunogold labelling to show that the binding of the myotoxic sPLA_2_ notexin was also to the plasma membrane [108,109] (Figure 19). There was no evidence of any internalization of notexin.

Binding was associated with the appearance of lesions in the plasma membrane, extensive hypercontraction, with the tearing of sarcomeres and the disruption of the plasma membrane. Intracellular mitochondria became swollen and floccular in appearance. The basal lamina remained structurally intact and satellite cells of both damaged and undamaged muscle fibres become activated [110,111]. The microcirculation, however, remained intact and capillary intermittency was reduced leading to increased blood flow in the damaged muscle [112].

**Figure 20:**
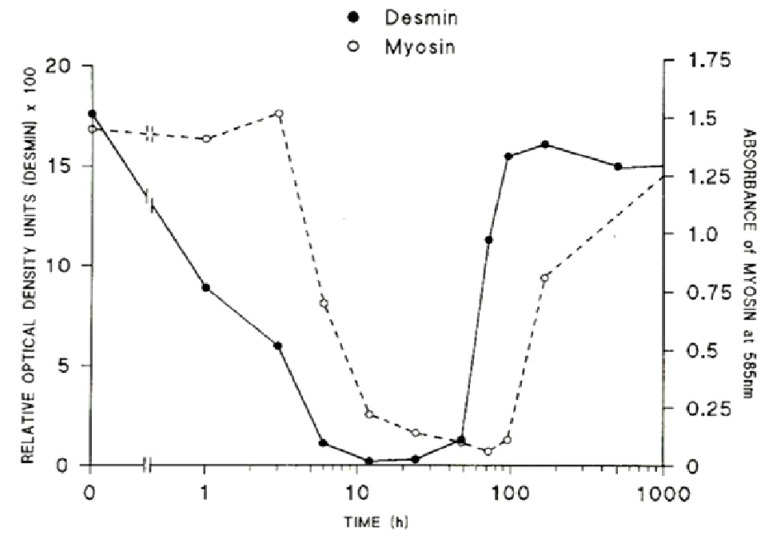
The relative rates of loss of desmin and myosin from muscles at various times after the inoculation of the venom of *Notechis scutatus*. (From Harris *et al*. 2003 [114]). Reproduced with permission from the Publisher.

The loss of the structural integrity of the plasma membrane accelerates the loss of ion homeostasis and the movement of the principal ions K^+^, Na^+^ and Ca^2+^ down their respective gradients. This leads directly to hyperkalaemia, and the elevation of [Ca^2+^]_i_. The sarcomeric proteins, desmin and titin (sometimes known as connectin) are rapidly destroyed, protein content falling by 50% within 1 and 3 h respectively [113,114]. The very rapid loss of desmin and titin that follows exposure to myotoxic venoms and myotoxic sPLA_2_ is significant. Titin is a highly elastic protein that is particularly important for the stabilisation of the sarcomere as it spans the structure between M-line and Z-discs. Desmin is similarly important as it forms a net around the Z-discs of adjacent sarcomeres and also links peripheral Z-discs to the cytoskeleton at the plasma membrane. The loss or lack of these proteins results in structural instability of the muscle fibres. Both proteins are very sensitive to the of Ca^2+^-activated, non-lysosomal, proteolytic enzymes (calpains) within the cell [115,116,117]. The contractile proteins myosin and actin are slower to degenerate, a 50% loss occurring by 6 and 9 h respectively (Figure 20).

Phagocytic cells that enter the degenerating muscle fibres appear to be primarily involved with the clearance of damaged mitochondria [113]. It is likely that this process of degeneration also involves the activation of the lysosomal, proteolytic enzymes, cathepsins B, L and H which are implicated in inflammatory muscle degeneration [118]. 

In summary, the data concerning the myotoxicity of many venom-associated Type 1 sPLA_2_s suggests that the sequence of events involved in the myotoxicity is the binding of the PLA_2_ to the plasma membrane followed by the hydrolysis of the lipids at the interface. The generation of lysophospholipids and free fatty acids creates an increased fluidity of the membrane leading ultimately to its tearing, the loss of ion-gradients, depolarisation, and the entry of calcium and hypercontraction of the myofilaments. Concurrently, the elevation of [Ca^2+^]_i_ leads to the up-regulation and activation of cPLA_2_, the hydrolysis of intracellular lipids and the release of fatty acids (including the pro-inflammatory arachidonic acid) into the cell interior. The combination of elevated [Ca^2+^]_i_, hydrolytic activity of the PLA_2_ and the presence of fatty acids is highly toxic to mitochondria and other sub-cellular structures such as sarcoplasmic reticulum and leads rapidly to the metabolic run-down of the cell and its ultimate death [119,120]. Recent observations on the myotoxic activity of the venoms of *Bothrops asper* and *Crotalus durissus terrificus* have shown that mitochondrial alarmins are released from exposed muscle and may contribute to the general pathology including the activation of the immune system and the inflammatory response [121]. 

Type 2b venom-derived sPLA_2_ homologues may be found in the venoms of a number of viperid snakes. These non-hydrolytic PLA_2_ homologues are myotoxic. Their toxicity makes clear that there is not an essential connection between hydrolytic and myotoxic activity. Lomonte *et al*. 1994 have shown that these myotoxic compounds possess … “a stretch of residues located at the C-terminal region of the molecule”… This stretch of residues (homology positions 112–129) together with residues Lys 36 and 38 is hydrophobic and …. “can interact with and disorganise the plasma membrane of cells” [122]. It is important to note, however, that many of these toxins are not specific myotoxins but general cytotoxins whose activity includes myotoxicity [122]. 

**Figure 21:**
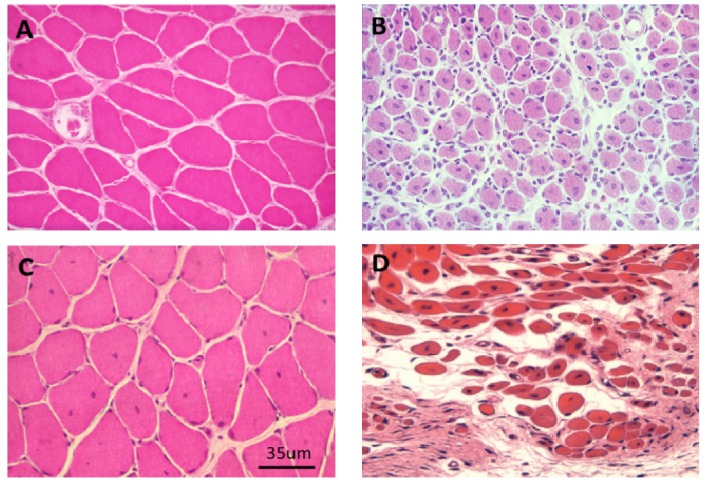
(**A**–**C**) Transverse sections of soleus muscles stained with H&E. A. control. B, C. Four and 28 days respectively after exposure, *in vivo*, to notexin, an sPLA_2_ from the venom of the Australian tiger snake, *Notechis scutatus*. Note the rapid growth of the muscle fibres and the continuing presence of centrally located myonuclei; (**D**) As above 28 days after exposure *in vivo* to the venom of the Fer de Lance, *Bothrops asper*, a viperid snake that causes extensive soft tissue necrosis (see Figure 5). Note the immature appearance of the regenerating muscle fibres and the extensive infiltration of connective tissue.

There have been several recent reviews concerning aspects of the normal development of skeletal muscle and its regeneration and the topic is not discussed here in any detail. The interested reader should refer directly to those reviews [123,124,125,126]. In brief, effective regeneration depends critically on the survival of the basal lamina tube within which regeneration proceeds, the invasion of inflammatory cells, the activation, within six 12 h, of the of the satellite cells, the maintenance of the microcirculation and the re-instatement of the motor innervation. Activated satellite cells in the damaged muscle fibres divide and, reinforced by other satellite cells migrating from undamaged muscle fibres (and possibly from connective tissue and endothelium), repopulate the empty basal lamina. By 24–48 h the satellite cells fuse to form multinucleated myotubes. New plasma membrane and basal lamina form and the old basal lamina is discarded. Following fusion the intermediate filamentous proteins vimentin, nestin and desmin become co-localised around the nascent Z-disc. By three days newly formed sarcomeres are established and by seven days muscle fibres are fully formed. At this stage, as long as the muscle fibres are innervated, they continue to grow to become fully mature at 10–21 days. In most respects the muscles are indistinguishable from undamaged muscles but, in rodents especially, the regenerated muscle fibres retain centrally located nuclei and in muscle fibre types rarely differentiate into the fast-twitch phenotype [127] (Figure 21).

Functional regeneration begins at three to four days when indirectly elicited action potentials can be recorded from most immature muscle fibres. Neuromuscular transmission is fully established by seven days and the contractile response of the muscle to indirect stimulation increases progressively thereafter to become similar to normal muscles by 21 days [96,97]. 

The process of regeneration of skeletal muscle in muscles damaged by both the crude venoms of viperid snakes and the relevant Group 2 (catalytic) and 2b (non-catalytic) sPLA_2_ homologues is essentially no different to that described above for Group 1 sPLA_2_s except that muscle regeneration is often slow, impaired and incomplete and the regenerated muscles are weak and heavily impregnated with connective tissue (Figure 21D). This is because many viperid venoms are rich in cytotoxic and haemorrhagic toxins. Myotoxic damage is often associated with significant local soft tissue damage involving, particularly, the microvasculature and the intramuscular nerves. Under these circumstances muscle regeneration is not efficient [128]. 

## 9. The Binding of sPLA_2_Types 1 and 2 to Excitable Membranes

Although it seems clear that the toxic sPLA_2_s target primarily the plasma membranes of excitable cells the nature of binding is still not fully established. The highly specific pharmacological nature of the toxic sPLA_2_s and the relatively low level of general toxicity of the catalytically active pancreatic sPLA_2_s suggest that the neuro- and myotoxic sPLA_2_s do not simply engage with membrane lipid substrates but also interact with some other receptor or acceptor prior to engaging with the natural lipid substrate. The distinction between receptor and acceptor is important. The term receptor, as used in pharmacology, refers to a specific site on a cellular macromolecule to which a transmitter, hormone, toxin or drug binds without itself being changed. As a result of binding a change in cellular activity is induced [129]. The key is that binding can be shown to result directly to a change in cellular behavior. If no clear change in cellular behavior can be recorded then the binding site is conventionally referred to as an acceptor. Numerous attempts have been made to identify membrane proteins that act as acceptors (and possibly receptors) for the neuro- and myotoxic sPLA_2_s. 

Oberg and Kelly used ^125^i-β-bungarotoxin to label membrane fragments from rat brain. They reported numerous binding sites in low density fragments of brain, and brain mitochondria but could not determine whether the binding sites comprised protein, carbohydrate or lipid [130]. McDermot *et al*. used [^3^H]-pyridoxylated β-bungarotoxin to label synaptosomes and synaptic vesicles from rat brain. They reported binding at relatively low affinity to a protein acceptor that was distributed widely in several membrane preparations including synaptic vesicles [131]. Othman *et al*. prepared [^3^H]-proprionated β-bungarotoxin and reported saturable binding with high affinity to an unidentified protein in the membranes of rat brain synaptosomes [132]. Rehm and Betz and Schmidt *et al*. used ^125^I-β-bungarotoxin to study binding to chick and rat brain membranes. Both reported specific binding to uncharacterised sites [133,134]. ^125^I-labelled ammodytoxin C from the venom of *Vipera ammodytes ammodytes* and ^125^I-labelled crotoxin have been shown to bind with high affinity to presynaptic nerve terminal membranes from *Torpedo marmorata*. The binding sites for the former are proteins with a mass of 20,000 Da and 70,000 Da; that for crotoxin is a protein with a mass of 48,000 Da. Ammodytoxin C also bound with lower affinity to a number of other proteins with masses of between 39,000 and 57,000 Da. None of the sites has been shown to relate to any pharmacological activity of the respective toxins [135]. Lambeau and his colleagues have studied the binding of a number of toxic sPLA_2_s to specific, identified, cloned and characterised “N” acceptors of 18–24 kDa, 36–51 kDa and 85 kDa from presynaptic neuronal membranes and “M” acceptors of 180 kDa isolated from myogenic cells [136]. There is currently no consensus that the identification of specific binding to neural tissues or to identifiable receptors/acceptors might be relevant to the expression of toxicity in intact tissue or in the whole organism. In particular, the “N” and “M” receptors have not been found in human tissues. 

The possibility that other such acceptors exist in neural, muscular or other tissues, is of great interest and a number of tantalising observations have been reported. One of the earliest was the interaction between venom-derived sPLA_2_s and K^+^ channels at the neuromuscular junction [86,87]. This was of interest because, as pointed out in section 7 above, the presumed binding appears to be independent of external Ca^2+^ and the pharmacological activity does not depend on hydrolytic activity. The binding has never been demonstrated morphologically and may not be specific. It is not clear that the association of venom-derived sPLA_2_s with K^+^ channels at the neuromuscular junction has any relevance to the neuro-myotoxicity seen in envenomed subjects. 

Vimentin, an intermediate filament protein, has also been identified as a potential acceptor for venom-derived sPLA_2_s. This is based on the observation that vimentin is partially externalized on the cell surface of apoptotic human T-cells where it acts as a target for a human group IIa sPLA_2_. The hydrolytic activity of the sPLA_2_ is enhanced and the association may enable its internalization [137]. The finding may be related to inflammatory events in the CNS but is unlikely to be related to the peripheral neuro-myotoxicity. Vimentin has never been identified as locating to the cell surface in either neural or muscular tissue; it is also developmentally regulated in both tissues and expression is suppressed in very early stages of maturation [138,139]. 

Two acceptors for sPLA_2_s have been identified in porcine tissues, both of which may be considered C-type multilectins. One acceptor, isolated from the cerebral cortex, is similar to the “M” receptor. The second, isolated from the liver, was recognized by anti-rabbit “M”-receptor IgG but was not related to that from the cerebral cortex [140]. 

Cytoplasmic proteins have also been identified as acceptors for sPLA_2_s. Examples include gamma and epsilon isoforms of a 14-3-3 protein [141]. It has been suggested that this interaction could explain—or contribute to—some of the neurotoxic effects of sPLA_2_s including the inhibition of neurotransmission and neuronal cell death. The process would clearly require the internalization of the sPLA_2_s at the motor nerve terminal. As discussed above (see Section 6) internalization is unlikely to be a major event in the peripheral nervous system. It might be a significant feature in the central nervous system with respect to endogenous sPLA_2_s. 

Similar reservations may be discussed with respect to the binding of neurotoxic sPLA_2_s to calmodulin, a Ca^2+^ sensor in many eukaryotic cells [142]. By using a number of mutant forms of the IIA sPLA_2_, ammodytoxin A, there was shown to be no correlation between toxicity and binding to calmodulin. Whether the binding of sPLA_2_ to calmodulin has any role to play in peripheral neuro-myotoxicity is unclear, primarily because of the need for the sPLA_2_ to enter the nerve terminal or muscle fibre.

Whatever the eventual outcome of these studies, the principle has been established that sPLA_2_s of snake venoms may act at different sites as either enzymes targeting phospholipids in cell membranes or as pharmacological ligands binding to a number of protein receptors/acceptors differentially distributed between different cell types. There may be as yet undetermined roles for these diverse targets of sPLA_2_s of snake venoms in the aetiology of neuro-myotoxicity in mammals but none have yet been determined. The studies may ultimately be found to be more relevant to the aetiology of neurodegenerative disease in the CNS in which endogenous phospholipases play a significant role [3,4,5,6].

## 10. The Treatment of Envenoming

The only specific treatment of envenoming in clinical situations is the administration of antivenom. Antivenoms are gamma immunoglobulins (IgGs) raised typically in horses or sheep following subcutaneous immunisation with sub-lethal doses of snake venoms. The antivenom might be raised against a single venom to produce monovalent antivenom or against the pooled venoms of several species of snake to produce a polyvalent antivenom. The venoms are usually taken from snakes known to be dangerous within a given country or identifiable geographical area. As a result antivenoms raised for use in one specific geographical region may be of very limited use in an entirely different region. Ideally a monovalent antivenom is used because the risk of serious adverse reactions is reduced, but that is only possible if the biting species is known beyond reasonable doubt. A polyvalent antivenom is used when the biting species is not known with certainty. Antivenoms should be used as soon as clinical signs of coagulopathy, neurotoxicity or soft tissue damage appear. This can occur within 30 min of a bite. If the use of antivenom is delayed the damage caused by the venom may not be reversed; at best the condition of the patient will be stabilised. In the case of neurotoxicity it has been stated: “Clinical experience shows that unless antivenom is given within 4 h of a bite by a snake causing presynaptic neurotoxicity most patients will continue to deteriorate and will require intubation and ventilation” [143]. In most impoverished rural areas across SE Asia, Africa and the Indo-Pacific bitten patients may not reach a competent clinical facility for many hours [144]. Even though appropriate antivenoms are very effective at neutralizing circulating venom the symptoms of envenoming may return as the antivenom is consumed and the release of venom from its depot at the bite site continues. Thus repeated administration is often needed. 

As well as these purely clinical problems there are numerous economic and logistical problems associated with antivenom use [145] and these and summarised below:

- In the poorer countries, where bites are common, antivenoms are often prohibitively expensive. This leads either to the death or prolonged suffering of victims denied access to antivenom or antivenoms being withheld until the patient is severely ill and any related tissue damage is irreversible.
- Antivenoms are raised in animals and thus adverse effects, including bronchospasm, anaphylactic shock, pyrogenic problems and delayed serum sickness are common.
- Considerable skill is required in the administration of antivenoms. They cannot be safely used in isolated rural clinics unless clinic staff have received specific training in respiratory management and the insertion of a venous line to deliver a controlled infusion of the antivenom.
- Suitable storage facilities for antivenoms are frequently unavailable in rural clinics and many are ill-quipped to deal with seriously envenomed patients.

These problems have given rise to a number of investigations into the use of alternative approaches to the management envenoming snake bites. There are three general strategies [146]. The first is to examine whether “native” plant-based remedies used in traditional medicine for the treatment of snake bite are truly effective [147]. The second is to examine the tissues of animals perceived to be relatively resistant to snake bite (for example, the numerous snakes known to be resistant to self-inflicted bites or bites by predatory snakes, the mongoose, the opossum and the hedgehog) in an attempt to isolate and characterise those tissue components that confer protection [24]. The third is to design synthetic or semi-synthetic drugs that are specific PLA_2_ inhibitors [6]. 

The development of plant-based remedies for major health problems has a long history in medicine. Notable examples are the way the use of extracts of the bark of trees of the genus *Cinchona* as a febrifuge by local South American tribal groups, led to extensive clinical studies on the powdered bark (Jesuit’s powder) during the 17th and 18th centuries. These studies demonstrated the efficacy of the material and led eventually to the isolation of cinchonine and quinine by, respectively, Gomez and Pelletier and Caventou during the early 19th century and the use of quinine as the treatment of choice for malaria for many decades [148]. Another example is the way the use of *Digitalis* sp. in the treatment of “dropsy” by village herbalists in rural England during the 18th century led ultimately to the isolation of digoxin by Nativelle in the mid 19th century and its incorporation into the management of congestive heart failure [149]. Features common to these two examples are the detailed clinical studies on Jesuit’s powder by Sydenham and Talbot, and by Withering on the use of extracts of *Digitalis* in the treatment of cardiac problems. In the majority of contemporary studies on herbal remedies used by native healers there is very little real evidence of clinical efficacy and little effort to generate such evidence. Moreover, the studies rarely consider that traditional practices and patterns of use of specific products often vary according to local cultures. Finally, most studies try unsuccessfully to translate observations made *in vitro* to clinical observations. Herbal remedies used in traditional forms of medicine would appear to offer little to the clinical management of envenomed subjects. Borges *et al*. have made a detailed discussion of many of these issues to which the interested reader is referred [150].

The natural resistance of many animals to the effects of natural venoms is of considerable biological interest. In most cases it would appear that there are circulating factors that neutralise the venom constituents of potentially dangerous snakes and thus confer true resistance. Thwin and Gopalakrishnakone [24] have made a detailed summary of animal sera and specific components isolated therefrom that exhibit anti-haemorrhagic, anti-neurotoxic, anti-myotoxic or anti-sPLA_2_ activity or inhibit the lethality of venoms in experimental animals. Protective factors or sera have been obtained from venomous snakes—*e*.*g*., *Trimeresus flavoviridis*, *Vipera palaestinae*, *Agkistrodon contortrix mokasen*, *Crotalus durissus terrifficus*, *Notechis scutatus*, non-venomous snakes—*e.g., Natrix tessellata*, *Python reticulatus* and Opossums and Mongooses. It is of relevance that sPLA_2_s are central to the toxicological aspects of envenomation as they are directly or indirectly involved not only with with neurotoxicity and myotoxicity but also with coagulopathies and inflammation. Thus identifying those factors in the sera of resistant species, determining the structural features elements that underpin the inhibitory activity and understanding their molecular biology offers potential for the development of novel therapeutic agents for the management of sPLA_2_-related disease, including envenoming and neurodegenerative and inflammatory diseases of the CNS. 

The third strategy, development of specific chemical inhibitors of PLA_2_s is an area of great excitement. For example, Thwin *et al*. [151,152,153] have generated a series of small polypeptides based on a phospholipase inhibitor protein (PIP) originally isolated from the serum of *Python reticulatus*. One polypeptide, identified as Peptide 10F, was of particular interest because it inhibited purified human synovial fluid sPLA_2_ in a dose dependent manner, probably by blocking the hydrophobic cleft that constitutes the active site [2,14] and also suppressed the production of both sPLA_2_ and matrix metalloproteinases from cultured, human, synovial fibroblasts activated by IL-1β. The results have clear implications for the treatment of rheumatoid arthritis in human patients. Chen *et al*. used a synthetic inhibitor of cPLA_2_ (arachidonyl trifluromethyl ketone) and demonstrated a reduction in both the onset and the progression of experimental autoimmune encephalomyelitis in mice in a dose dependant manner [154]. It has also been shown that the selective inhibition of sPLA_2_ by a small synthetic peptide, CHEC-9, protected cultured SY5Y cells from induced stress and also inhibited the differentiation of HK60 cells. The data suggest that the selective inhibition of both s- and cPLA_2_ could have a major impact on both cell death and the activation of the inflammatory process [155]. Similar studies on the use of PLA_2_ inhibitors for the control of inflammatory processes in other clinical situations have already reached the stage of clinical trial [2]. 

The future treatment of envenoming snake bite could be dramatically improved with the development of specific inhibitors of sPLA_2_s, designed to enter the peripheral circulation rapidly following administration but excluded from the CNS. They could offer safe, easily managed, first line protection against circulating venom sPLA_2_s prior to the patient reaching a major referral centre where skilled medical assistance is available.

## 11. The Problem of Pain

Many patients experience pain following an envenoming snake-bite. The pain may be confined to the bite site, or involve the bitten limb, or be reported as generalised myalgia. Pain may also reflect lymphadenitis, affecting lymph nodes in the groin, the axilla or the abdomen depending on the site of the bite. On other occasions—especially following bites by kraits—the pain may be confined to the loins. 

Pain at the bite site is usually transient and relatively easily managed with minor analgesics, or even placebo, but more generalised pain may be severe and require NSAIDs and opioids [71,156]. It is clear that the origin of pain is multi-factorial. Contributory factors include the physical trauma of the puncture wound; the promotion of the release of histamine and 5HT from mast cells by the sPLA_2_ toxins; the invasion of inflammatory cells and the release of K^+^ and the cytokines IL-1 and TNF from either plasma or damaged soft tissue. The disruption of the muscle sarcolemma and the increase in [Ca^2+^]_i_ may lead directly to the activation of cPLA_2_s, the release of arachidonic acid and the production of prostaglandins and eicosanoids. To this complicated situation must be added the problems caused by bite-site cutting and the use of over-tight ligatures as practiced in so many parts of the world, a problem that persists in spite of numerous attempts to change established practice in rural communities.

There is very little experimental work on the biological basis of venom-related pain and clinical management is typically designed to provide symptomatic relief. There is a need for an expansion of work in this general area, alongside a pressing need to effect change in the early management of snake bite. One possible route would be to recognise the role played by local healers and provide proper training and financial incentives to those healers prepared to abandon inappropriate “first aid” measures such as the use of ligatures and bite-site cutting and to adopt instead evidence-based services to patients such as immobilisation and constriction bandaging.
